# Perfluoroaryl and Perfluoroheteroaryl Reagents as Emerging New Tools for Peptide Synthesis, Modification and Bioconjugation

**DOI:** 10.1002/chem.202103305

**Published:** 2021-12-02

**Authors:** William D. G. Brittain, Christopher R. Coxon

**Affiliations:** ^1^ Department of Chemistry Durham University South Road Durham DH1 3LE UK; ^2^ EaStChem School of Chemistry The University of Edinburgh Joseph Black Building David Brewster Road Edinburgh EH9 3FJ UK

**Keywords:** bioconjugation, fluorine, functionalisation, peptide, perfluoro(hetero)aromatic

## Abstract

Peptides and proteins are becoming increasingly valuable as medicines, diagnostic agents and as tools for biomedical sciences. Much of this has been underpinned by the emergence of new methods for the manipulation and augmentation of native biomolecules. Perfluoroaromatic reagents are perhaps one of the most diverse and exciting tools with which to modify peptides and proteins, due principally to their nucleophilic substitution chemistry, high electron deficiency and the ability for their reactivity to be tuned towards specific nucleophiles. As discussed in this minireview, in recent years, perfluoroaromatic reagents have found applications as protecting groups or activating groups in peptide synthesis and as orthogonal handles for peptide modification. Furthermore, they have applications in chemoselective ‘tagging’, stapling and bioconjugation of peptides and proteins, as well as tuning of ‘drug‐like’ properties. This review will also explore possible future applications of these reagents in biological chemistry.

## Graphical abstract

Perfluoroaromatic and perfluoroheteroaromatic reagents have emerged in recent years as powerful reagents for peptide and protein modification. Dominated by their regio‐ and chemoselective nucleophilic aromatic substitution chemistry under mild conditions, they have been used in peptide synthesis, tagging, cyclisation, stapling and protein bioconjugation.

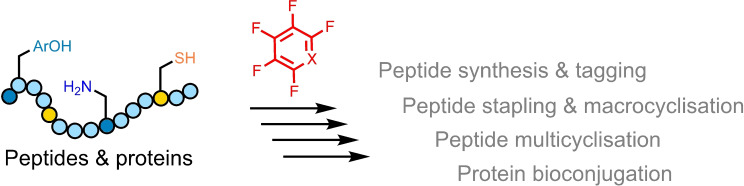

## 1 Introduction

Interest in peptides has increased significantly over recent decades, largely due to recognition of their potential therapeutic applications in combination with a growing tendency for drug‐discovery to move away from small flat compounds. Peptides can be highly potent and target‐selective APIs, with improved safety profiles over traditional small molecules and this has seen them find increasing clinical approval rates across a range of medical areas.^[1]^ Consequently, methodologies to access unnatural peptide architectures or to conduct late‐stage modification of peptides for example, to enhance drug‐like properties, has become increasingly important.^[2]^ A major challenge in peptide drug development is their sensitivity towards enzymatic degradation, potentially diminishing their efficacy. Indeed, the introduction of modifications and unnatural residues has shown that this propensity for enzymatic degradation can be subverted.^[3]^ In addition, late‐stage modification or bioconjugation approaches mean that large libraries of structurally diverse peptides can be readily accessed from a common starting peptide, thus, greatly reducing time taken for drug screening.^[4]^

Perfluoroaromatic and perfluoroheteroaromatic compounds (all hydrogen atoms replaced by fluorine) have become an increasingly popular tool in peptide modification and synthesis. This is for good reason, perfluoroaromatic compounds have been shown to be highly versatile moieties, participating in a range of transformations, and possessing highly unusual chemical properties themselves. Perfluoroaromatic compounds are well known to undergo aromatic substitution reactions with a variety of nucleophiles.^[5]^ Due to the electron withdrawing nature of several fluorine atoms, the aromatic rings within perfluoroaromatic compounds are extremely electron‐poor. This lack of electron density in the π framework in combination with the ability of flourines to act as leaving groups means that S_*N*_Ar processes are highly favoured. This has led perfluoroaromatics to be used as protecting groups,^[6]^ for bi‐aryl modification,^[7]^ macrocycle synthesis^[8]^ and in polymer chemistry,^[9]^ where highly controllable reactions are advantageous. The propensity to react with nucleophiles makes this class of compound a perfect candidate for peptide modification and bioconjugation.

This review provides a companion piece to the excellent general reviews of arylation and conjugation of peptides.^[10]^ It presents an up‐to‐date synopsis of the uses of perfluoroaromatic and perfluoroheteroaromatic compounds in unnatural amino acid synthesis, peptide modification and bioconjugation. It looks to highlight a range of synthetic methodologies that have been developed to incorporate perfluoroaryl functionality into peptides and also the ways in which perfluoroaryl groups have been used as reactive handles for further peptide modifications. Finally, some future directions are discussed.

## 2 Peptide Synthesis

Perfluoro(hetero)aromatic moieties can be incorporated into peptides through the synthesis of bespoke perfluoroarylated building blocks. This section will discuss the routes to incorporate perfluoroaromatic groups into amino acids and their subsequent incorporation into peptides.

### 2.1 Synthesis of perfluoroarylated building blocks

**Scheme 1:**
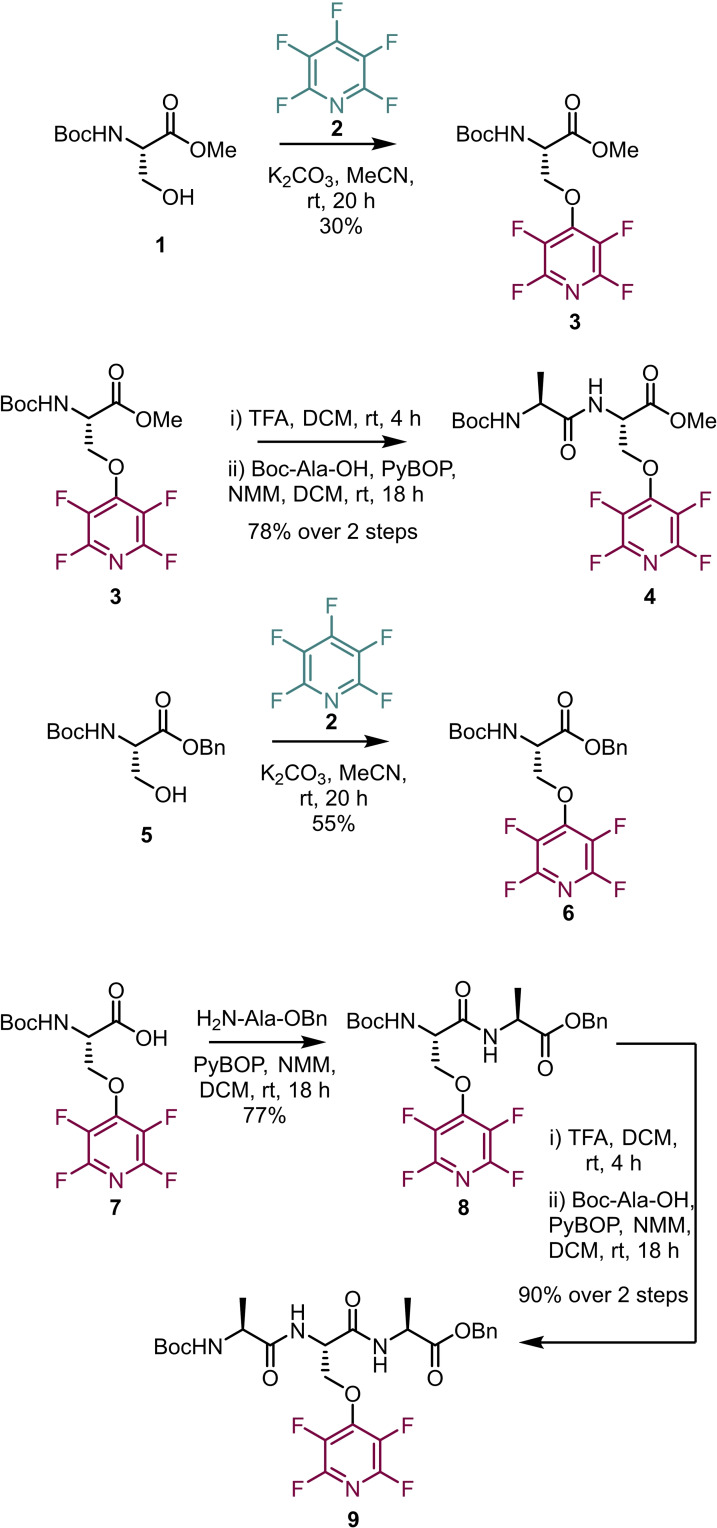
Synthesis of a tetrafluoropyridine‐containing amino acid and a tripeptide.

The propensity for perfluoroaromatics to undergo S_*N*_Ar processes lends itself to amino acid synthesis through a substitution approach with amino acids that contain nucleophilic side chains. The first use of this strategy was reported by Cobb and co‐workers in 2013, in which a *O*‐(4‐tetrafluoropyridyl)‐serine was prepared in 30 % yield by treatment of a protected serine with pentafluoropyridine (PFP) **2** (Scheme 1).^[11]^ *N*‐terminal solution phase peptide coupling was then demonstrated in a two‐step sequence with Boc‐Ala‐OH in 78 % overall yield. A second amino acid was synthesised this time using a Boc/benzyl ester protected serine **6** in 55 % yield. Benzyl deprotection, N‐terminal elongation, followed by coupling with Boc‐Ala‐OH was carried out to give the tripeptide **9** in 90 % overall yield.

**Scheme 2:**
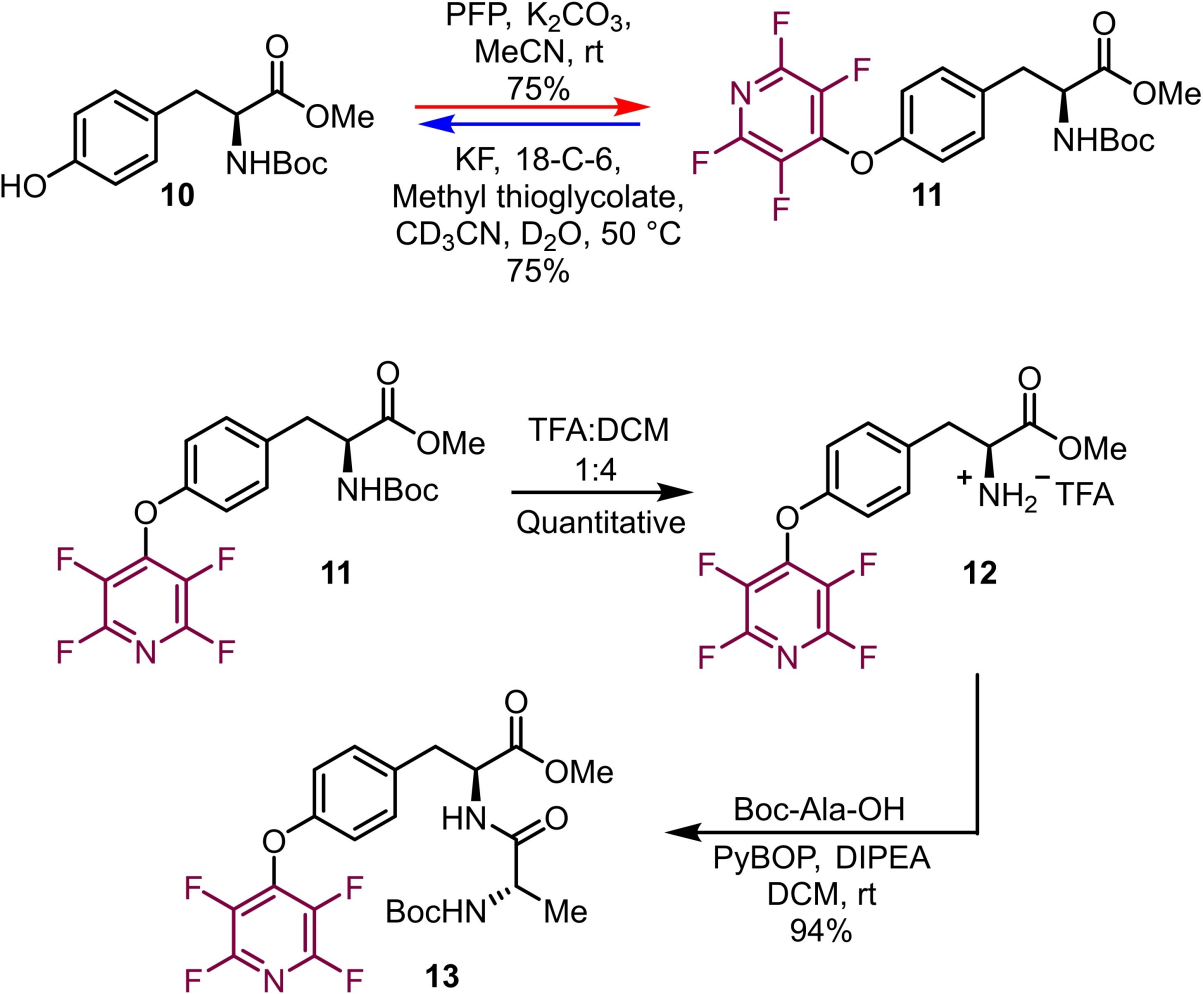
Synthesis, deprotection and peptide coupling of a tetrafluoropyridyl tyrosine amino acid.

This report was followed up on in 2019,^[6]^ as part of a larger study on the use of tetrafluoropyridyl moieties as protecting groups for phenols for example tyrosine (Scheme 2). Boc‐Tyr‐OMe **10** was treated with PFP **2** in the presence of potassium carbonate to generate the tetrafluoropyridyl‐appended amino acid **11** in 75 % yield. It was demonstrated that this perfluoroheteroaromatic tyrosine was compatible with peptide synthesis. Subsequent Boc deprotection followed by coupling with Boc‐Ala‐OH successfully generated dipeptide **13** in 94 % yield. This finding shows that the attachment of fluoropyridyl moieties to tyrosine is reversible and this could be of interest in peptides where dynamic properties are desirable.

**Scheme 3:**
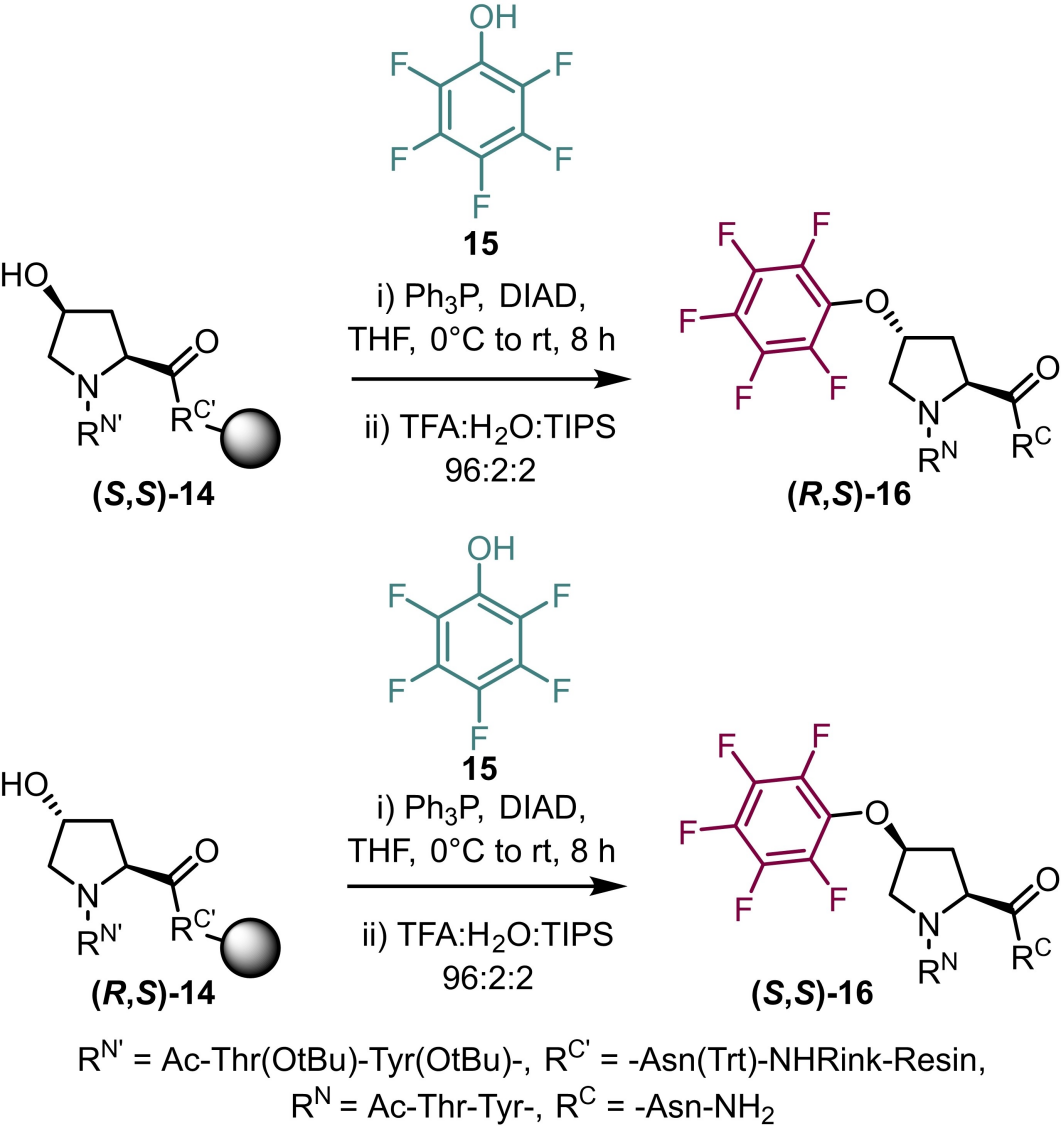
Synthesis of pentafluoroaryl‐ether prolines on solid phase using a Mitsunobu approach.

Another amino acid that contains a nucleophilic group is hydroxyproline (Hyp) and in 2013 Zondlo and co‐workers published an extensive report on 4‐substituted prolines and their effects on peptide conformation.^[12]^ Substituted prolines have been demonstrated to influence the conformation of the proline ring itself. For example, 4‐fluoroprolines have been show induce ring pucker through a *gauche* effect due to the electron‐withdrawing effect of the fluorine, highly influencing the *endo*/*exo* ring equilibrium and, therefore, the prolyl *cis*‐*trans* populations. In order to understand how 4‐position subsitution can have dramatic effects on proline ring and peptide conformation, Zondlo and co‐workers prepared a vast library of substituted prolines. As part of this study a Mitsunobu approach on solid phase was developed and applied to a range of nucleophiles (Scheme 3). Using this approach short peptides containing pentafluoroaryl ethers were synthesised **(*R***, ***S*** **)‐16** and **(*S***, ***S*** **)‐16**. Taking advantage of the spin active nature of fluorine‐19 as a highly sensitive NMR‐active nuclei, Zondlo was able to employ the technique to measure the prolyl bond *cis*/*trans* ratio.

**Scheme 4:**
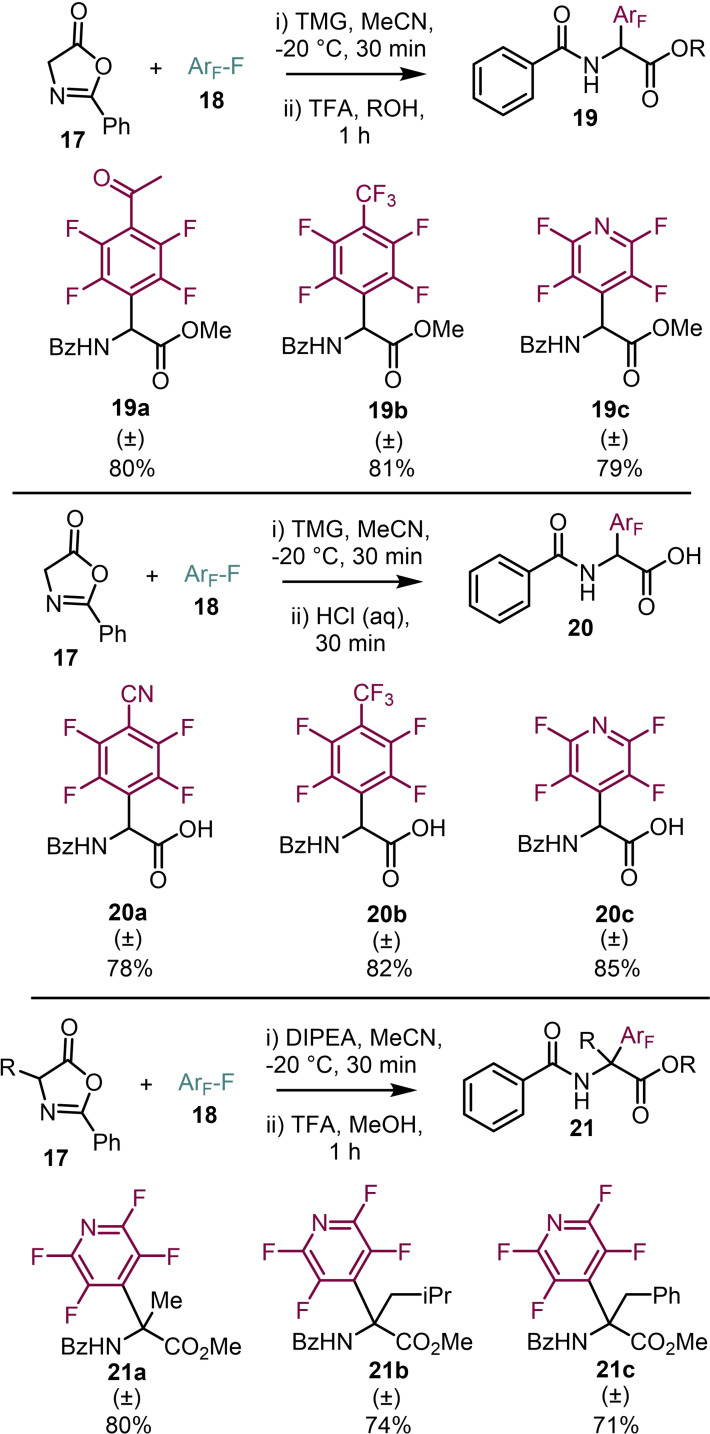
Synthesis of perfluoroaryl amino acids.

Teegardin and Weaver developed methodology to perfluoroarylate and then ring‐open oxazolones to access perfluoroaromatic amino acids where the perfluoroaryl group is attached directly to the amino acid α‐carbon back bone (Scheme 4).^[13]^ Treating 2‐phenyloxazol‐5(4*H*)‐one **17** with the desired perfluoroaromatics **18** in the presence of base led to arylation of the oxazolone, which was predisposed to ring opening. This could then be esterified with catalytic TFA in dry alcohol to give the protected amino acids **19 a**–**19 c**. By modification of the esterification procedure by using aqueous HCl the free carboxylic acids **20 a**–**20 c** were obtained. As part of the study, it was demonstrated that the methodology was also applicable to C4 substituted oxazolones to generate amino acids with quaternary carbon centres and a range **21 a**–**21 c** of protected amino acids were isolated.

**Scheme 5:**
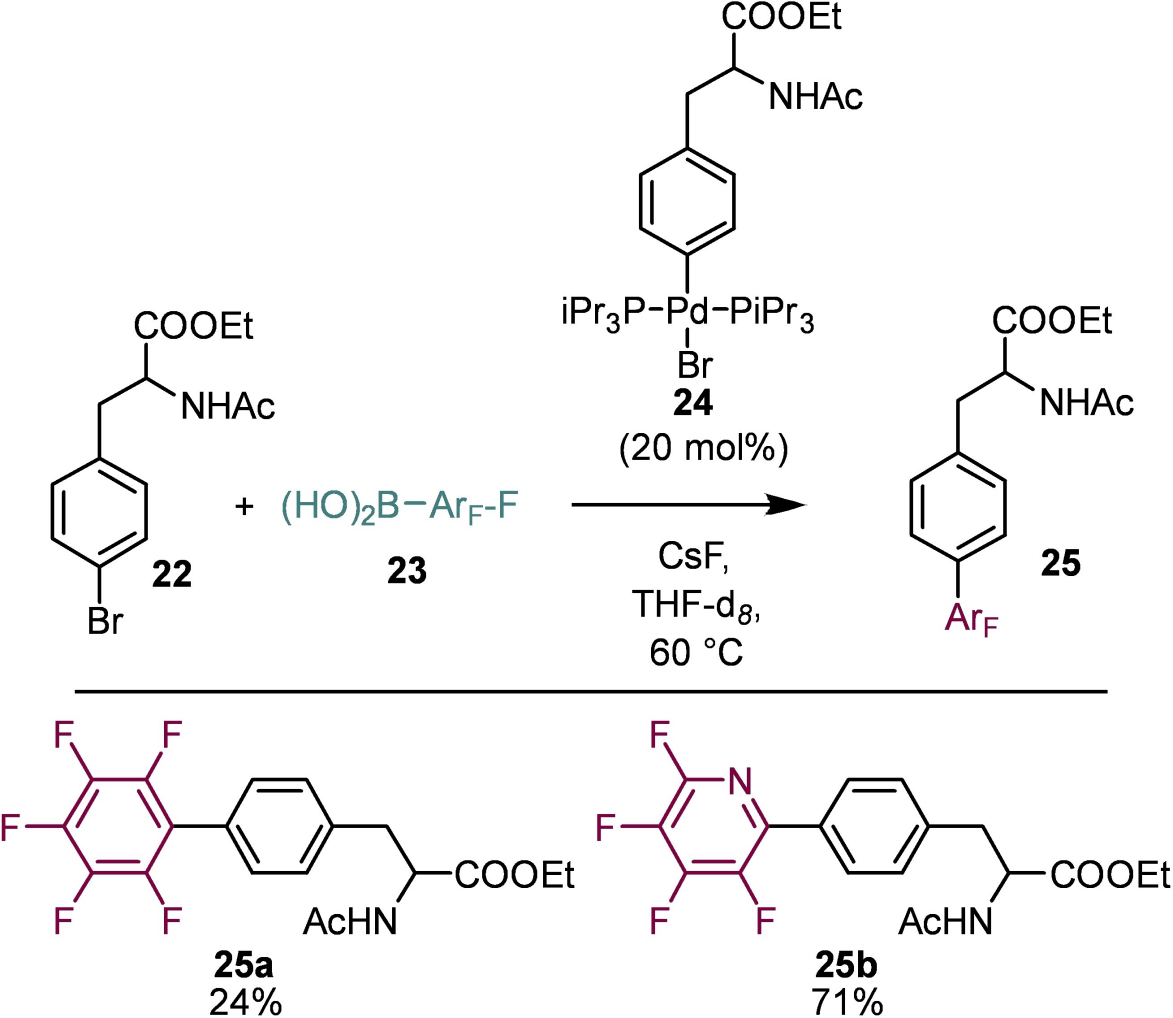
Synthesis of bi‐aryl amino acids.

In 2017 Herrmann and co‐workers disclosed the synthesis of bi‐aryl amino acids with perfluoroaryl functionalities (Scheme 5).^[14]^ Cross‐coupling methodologies have become the cornerstone in the synthesis of bi‐aryl amino acids and in Herrmann's report, a Suzuki cross‐coupling approach was taken.^[15]^ By treatment of compound **22** with [Pd(PiPr_3_)_2_] a highly active catalyst (**24**) was formed, this in combination with caesium fluoride allowed cross‐coupling to occur to access highly fluorinated bi‐aryl amino acids **25 a** and **25 b**.

### 2.2 Applications of perfluoroaryl groups in further functionalisation

The highly electron‐poor nature of perfluoroaryl groups affords their ability to stabilise anions and act as good leaving groups. For example, pentafluorophenyl esters are very reactive towards nucleophiles, allowing for nucleophilic substitution reactions to be conducted under mild conditions. Therefore, pentafluorophenyl esters have been employed to generate amides,^[16]^ esters^[17]^ and cross‐coupling products.^[18]^ Due to the mild conditions this approach has seen much use in the synthesis and modification of peptides and bio‐scaffolds.

**Figure 1:**
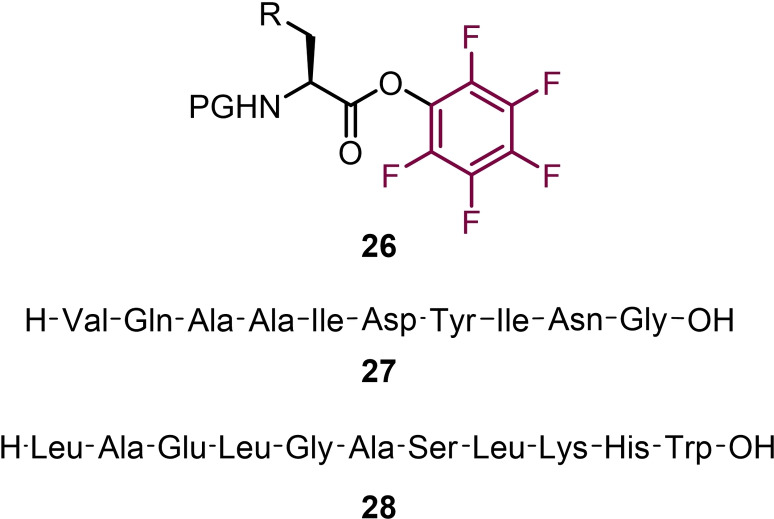
Synthesis of peptides on solid phase using pentafluorophenyl esters.

Pentafluorophenyl esters are applicable to solid phase peptide synthesis and this was first demonstrated by Atherton and Sheppard in 1985 (Figure 1).^[19]^ By using preformed Fmoc‐amino acid pentafluorophenyl esters **26** they were able to synthesise the acyl carrier protein 65–74. They found that the use of DMF and HOBt led to facile amide bond coupling and allowed the preparation of the desired decapeptide with the crude peptide displaying high purity (>90 %). In 1987 a follow‐up was published by Sheppard and co‐workers, which focused on optimisation of reaction conditions to use Fmoc‐amino acid pentafluorophenyl esters on solid phase.^[20]^ Using the decapeptide **27** and dodecapeptide **28** a range of reactions and conditions were trialled. It was found that pentafluorophenyl esters were easy to prepare for the vast majority of Fmoc‐amino acids (apart from Ser(tBu)‐OH and Thr(tBu)‐OH) and were easy‐to‐handle crystalline solids. For amide coupling using DMF as solvent and with HOBt as a catalyst. This first report laid the bedrock for which others would build upon in other uses of fluorophenyl esters in peptide modification.

In 2021, it was reported that pentafluoropyridine can also be employed to synthesise amide bonds.^[21]^ By mixing a carboxylic acid with pentafluoropyridine it was shown that the corresponding acyl fluoride could be readily generated. By subsequent addition of an amine, the amide could be generated in good to excellent yield across a wide range of examples.

**Scheme 6:**
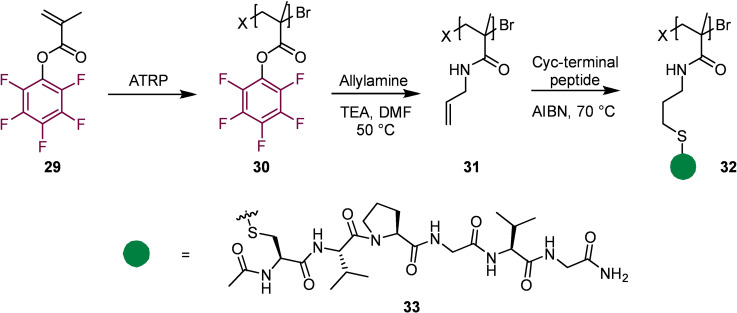
Synthesis of peptide appended polymers.

An example of the use of pentafluorophenyl esters in peptide conjugation was reported by Klok and co‐workers in 2011 (Scheme 6).^[22]^ Employing a pentafluorophenyl ester in atom transfer radical polymerisation (ATRP) gave a poly(pentafluorophenyl methacrylate) polymer, which was subsequently modified to give the allyl appended polymer **31**. This polymer was then used in a thiol‐ene “click” reaction to attach a peptide to make a highly defined peptide‐polymer conjugate **32**. Such conjugates have many applications in, for example, “smart” materials and in peptide drug delivery.

**Scheme 7:**
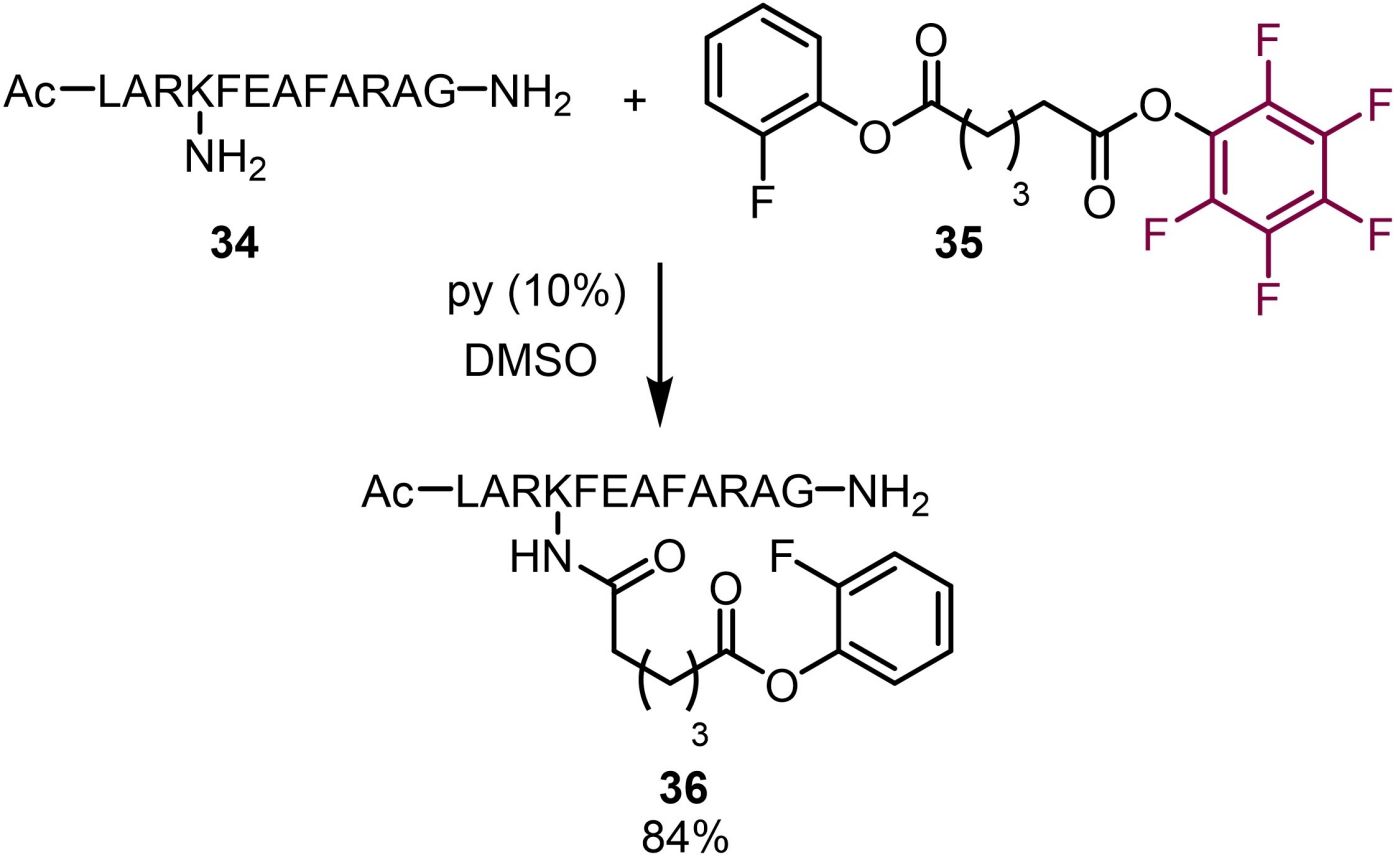
Peptide conjugation using fluorophenyl esters.

In 2012 Baltzer and co‐workers reported the synthesis of mixed pentafluorophenyl and ortho‐fluorophenyl esters and their use in peptide modification (Scheme 7).^[23]^ By employing two different fluorophenyl esters it was possible to obtain sequential amide bond formations due to the differences in reactivity. By mixing the mixed ester **35** with peptide **34** in the presence of base the conjugate **36** was isolated in 84 % yield. Using the ortho‐fluorophenyl ester‐peptide **36** they were then able to conduct a second peptide‐conjugation reaction to access large multi‐component peptides.

**Scheme 8:**
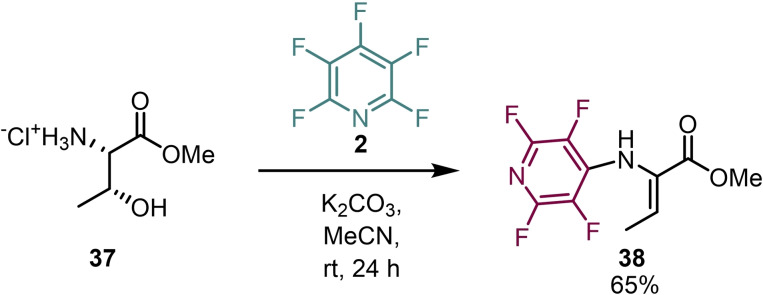
Synthesis of dehydrobutyrine derivatives.

Apart from being used as leaving groups for nucleophilic substitution reactions, perfluoroaromatics have been used to access dehydro‐amino acids (Scheme 8). Webster et al. demonstrated that the treatment of threonine with pentafluoropyridine afforded N‐tetrafluoropyridyl‐dehydrobutyrine **38**, via an E1cb‐type mechanism.^[24]^

## 3 Peptide Modification Using Perfluoroaromatic Reagents

The S_*N*_Ar arylation of peptide side chain nucleophiles by perfluoroaromatics provides a route to install a useful chemical handle for further chemical modification and for the transformation of linear unstructured short peptides into defined secondary structures for example macrocycles and helices. These specific applications depend on the nature of the perfluoro(hetero)aromatic reagent and its particular S_*N*_Ar reactivity profile.

### 3.1 Peptide perfluoroarylation

**Scheme 9:**
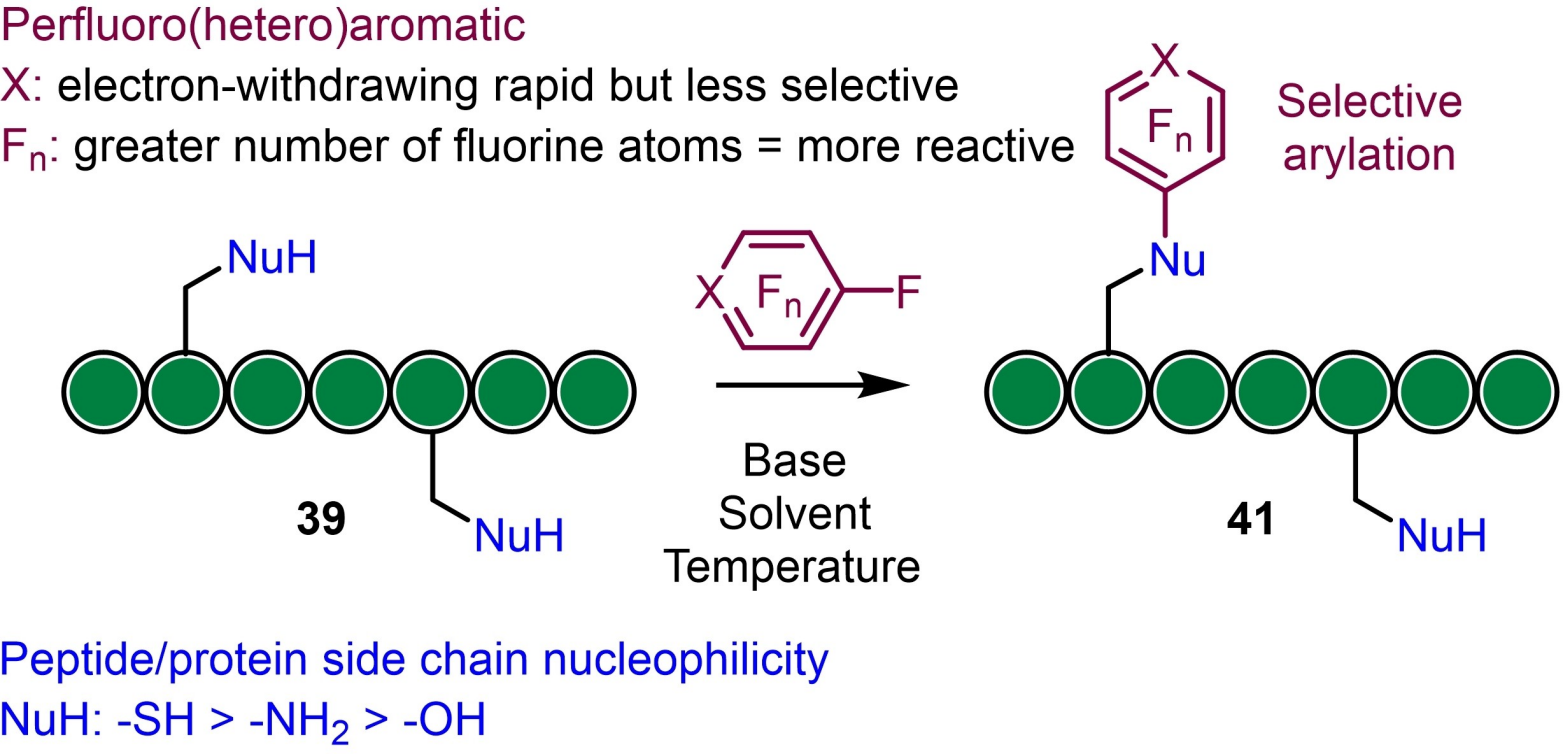
General approaches to controlling peptide perfluoroarylation.

Perfluoroheteroaromatics for example pentafluoropyridine tend to have broad reactivity towards nucleophiles in polar organic solvents (e. g. DMF) and reaction with unprotected peptides can afford multiple arylation products at, for example, cysteine thiols, tyrosine hydroxyl groups and free amines; whilst the less reactive hexafluorobenzene (**40**) tends to only react with cysteine thiols under mild conditions (Scheme 9).^[25]^ Used in excess, tetrafluoropyridazine (more electrophilic than pentafluoropyridine **2**) was found to afford complete arylation of tyrosine and the peptide N‐terminus in the ‘tagging’ of oxytocin. This was shown to increase resistance to chymotrypsin hydrolysis.^[25]^

The reactivity and chemoselectivity of pentafluoropyridine **2** can be controlled by the use of 2,2,2‐trifluoroethanol as a solvent, affording mild and clean reaction of pentafluoropyridine with cysteine residues in the presence of unprotected free amines and phenols (e. g. Lys, Tyr), whilst hexafluorobenzene was unreactive under these conditions.^[26]^ This was presumed to be due to the ability of TFE to solvate *O*‐ and *N*‐centered nucleophiles, reducing their apparent nucleophilicity over larger diffuse thiols. Alternatively, the reduction in the reactivity of the hexafluorobenzene/pentafluoropyridine in TFE (compared with DMF) could also be due to the Meisenheimer‐Jackson intermediate not being significantly stabilised during the S_*N*_Ar arylation reactions.

**Figure 2:**
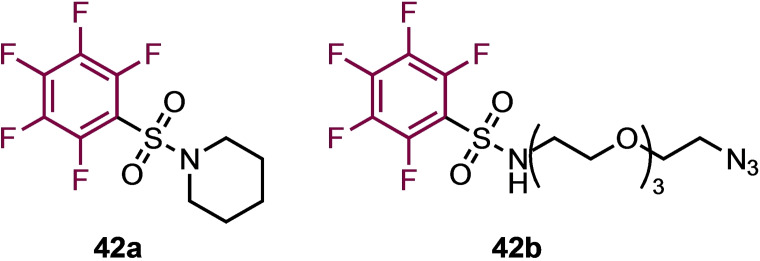
Compounds employed for selective labelling experiments.

The Diness Group, explored the reactivity and selectivity of S_N_Ar reactions between fluorobenzene derivatives and biologically relevant nucleophiles by a ‘two dimensional rational tuning’ approach.^[27]^ By altering the electron‐withdrawing ability of a phenyl *para*‐substituent and the number of fluorine atoms attached to the phenyl ring, reactivity towards (N‐Boc)‐Cys‐OH was probed in either, phosphate buffered saline (PBS) and acetonitrile at various pHs, or in a mixture of DMF and N,N′‐diisopropylethylamine (DIPEA). In general, the reactivity was found to be sensitive to the electron donating/withdrawing nature of the *para*‐substituent, and removal of fluorine atoms from the ring attenuated the reactivity. This led to the design of a highly reactive pentafluorophenyl sulfonamide **42 a** (Figure 2) that was found to be selective for cysteine arylation over other competing amino acids in a model peptide. Indeed, a further analogue **42 b** containing PEG‐azide (Figure 2) was shown to selectively label enhanced green fluorescent protein (eGFP) and human serum albumin, allowing fluorescent labelling; whilst reactivity‐tuned variants could also covalently label and inhibit cysteine proteases over serine proteases (at cysteine thiols) in a site‐selective and activity‐dependent (only when fully folded) manner.

**Scheme 10:**
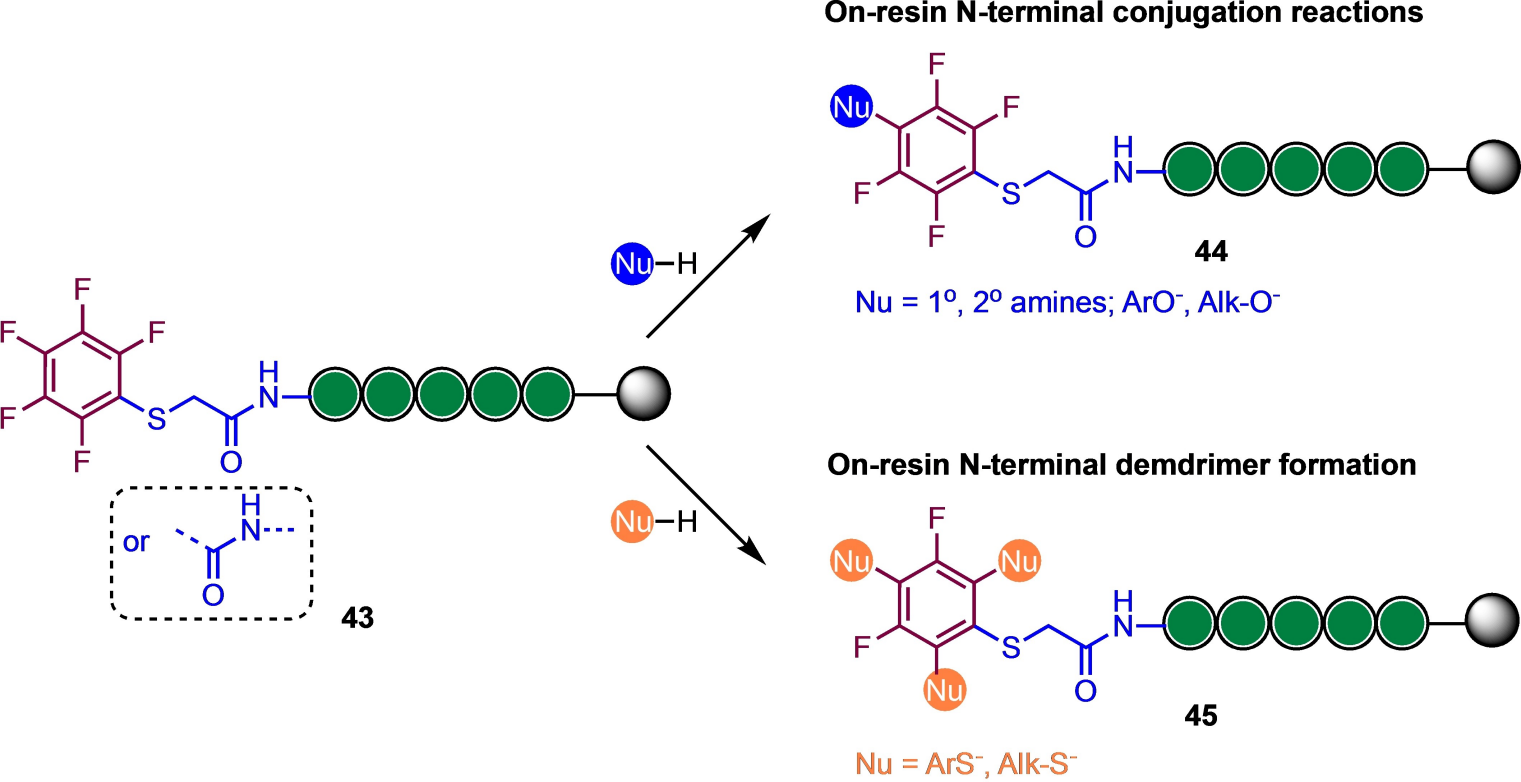
On‐resin approaches to peptide N‐terminal conjugation or dendrimer formation via perfluoroarylation. Based on Ref. [28].

Alapour and co‐workers investigated a reverse strategy ‐ incorporating the perfluoroaryl group into the peptide backbone for subsequent S_*N*_Ar functionalisation (Scheme 10).^[28]^ Here, they studied reactivity and site specificity of pentafluoroacetic acid and pentafluorothiophenol (attached via an iodoacetamide precursor) as useful new perfluoroaromatic linkers for on‐resin peptide diversification and demonstrated that these linkers are of approximately equivalent reactivity and can undergo S_*N*_Ar reaction with a range of nucleophiles (*S*‐, *N*‐ and *O*‐centred) using NMP as a solvent and DBU as a base in a relatively short (1 h) reaction time. Multiple *ortho*‐ and *para*‐substitutions were observed with some thiol nucleophiles, which gives access to a possible new strategy for peptide branching and dendrimer preparation.

### 3.2 Peptide stapling and macrocyclisation

Constraining linear peptides into defined secondary structures through macrocyclization or ‘stapling’, has become a leading area for research into new bioactive drug modalities. Macrocyclic/stapled peptides can access previously ‘undruggable’ targets with increased affinity, cellular penetration and resistance to proteolytic breakdown, and this has stimulated significant interest into new mild and regio‐/chemoselective ways to obtain such architectures.[^2d^, ^29^]

**Scheme 11:**
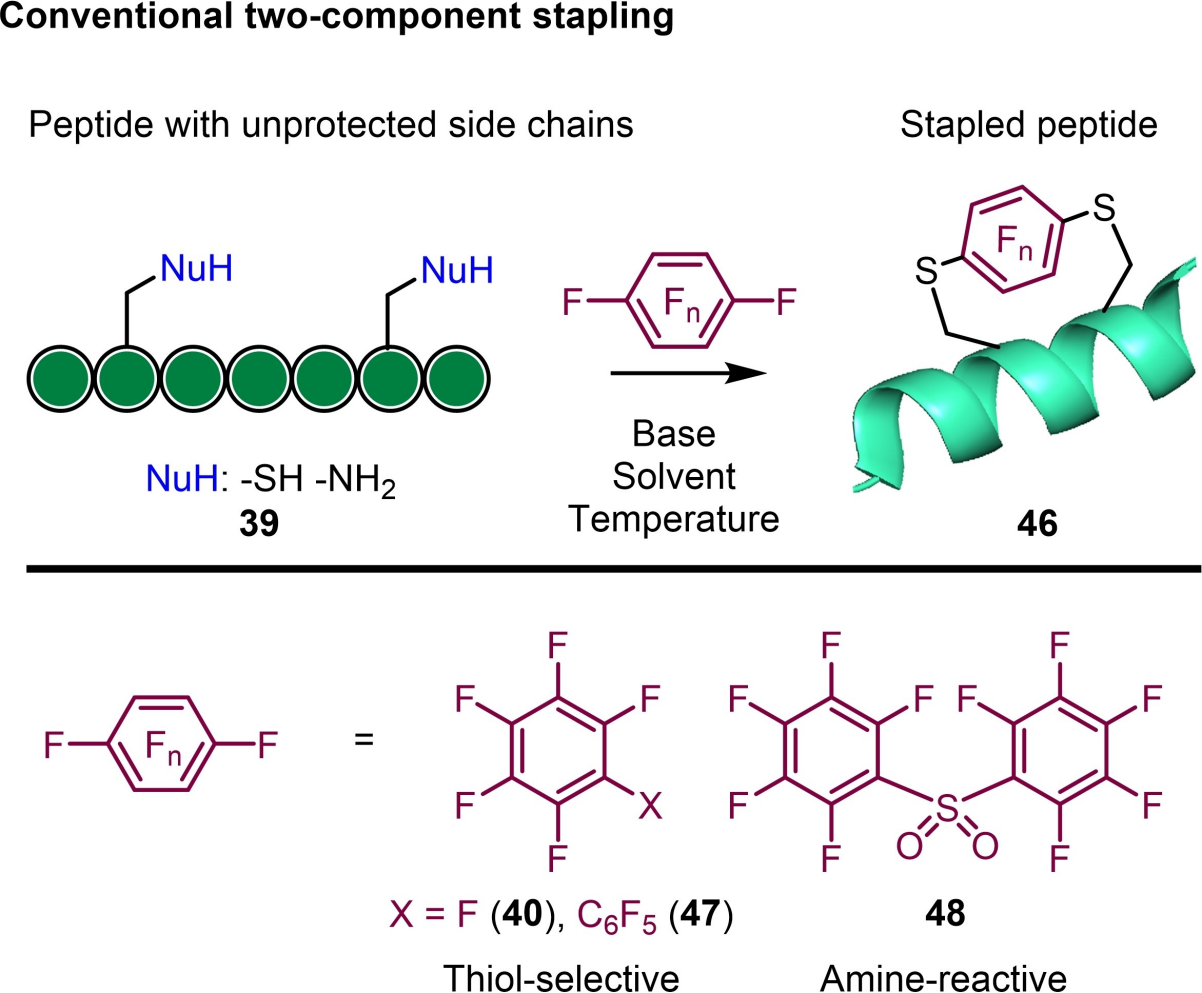
General approaches to two‐component peptide stapling by perfluoroaromatic reagents.

Hexafluorobenzene, is of relatively intermediate reactivity, but unlike pentafluoropyridine, readily undergoes successive regioselective 1, 4‐disubstituion (S_N_Ar) reactions (Scheme 11),^[5]^ particularly with thiolate nucleophiles. This makes it a useful reagent for disulfide cross‐linking chemistry in peptide and protein systems.^[30]^ The incorporation of two cysteine residues with relative *i* and *i+4* spacing into a peptide with a tendency to adopt an α‐helical conformation preorganises the thiolates on the same face of the helix. In basic buffer solution, hexafluorobenzene undergoes chemoselective two‐component cross‐linking of α‐helical peptides, and the resulting stapled peptides exhibited increased helicity and resistance to proteolytic degradation.

Decafluorobiphenyl (**47**) behaves similarly to hexafluorobenzene, undergoing a regioselective 4, 4‘‐double thiol‐fluoride substitution (Scheme 11), and providing a longer crosslinking motif, well suited to *i*, *i+7* disulfide crosslinking.^[30]^ Interestingly, the perfluoroaryl crosslinking of disulfides in transportan‐10^[31]^ and Pt(IV) prodrug peptides for glioblastoma treatment^[32]^ was also found to increase blood‐brain barrier penetration in models, and may be utilised in future for improved targeting of notoriously challenging CNS targets.

**Scheme 12:**
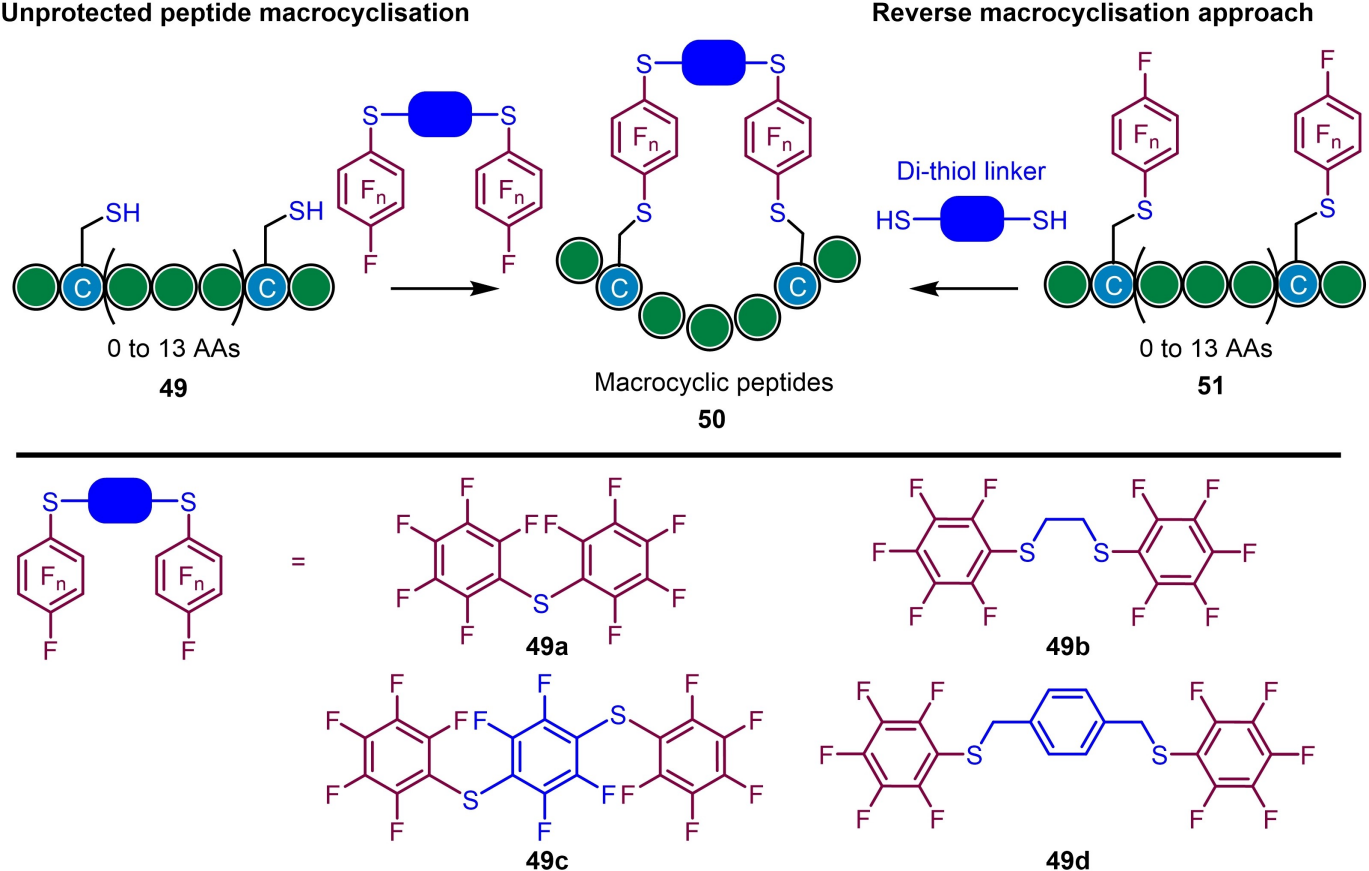
General approaches to peptide macrocyclisation using perfluoroaromatic‐based reagents. Note, peptide sequences shown are for illustrative purposes only. Based on Refer. [33].

Hexafluorobenzene and related thiol‐substituted pentafluorophenyl reagents have also been utilised in the side chain macrocyclization of non‐helical peptides, directly cross‐linking reduced disulfides (from *i*, *i*+1 to *i*, *i*+14) in an analogous manner to the above.^[33]^ In addition, a reverse approach was demonstrated, in which each cysteine was individually arylated (monosubstitution) with a non‐bridging perfluoroaryl group and then commercially available dithiol linkers were used for subsequent macrocyclization to afford diverse peptide libraries (Scheme 12).

This transformation has since been employed in several different peptide systems under related conditions, typically employing DMF as solvent and either TRIS‐base, Et_3_N or DIPEA as base, with reaction times ranging from 30 min up to 18 h.^[34]^ In one example, the reaction of hexafluorobenzene **40** with N‐acetyl cysteine was explored and optimised using a 2‐level factorial design experiment (varying: reaction time, temperature, concentration and equivalents of base/thiol) and monitoring the reaction outcomes by the distinct spectra obtained using ^19^F NMR analysis of crude mixtures.^[35]^ Application of the optimal mild conditions (DMSO/Cs_2_CO_3_, 21 °C or MeCN/DBU, 21 °C) to a model unprotected peptide system rapidly (<1 minute and < 1 h, respectively,) afforded the quantitative disubstitution (stapled) product. The reaction was also possible on‐resin (DMF, DIPEA, 21 °C, 18 hours), making this applicable to solid phase modification.

The thiol‐fluoride substitution reaction also proceeds smoothly using cysteine ‘analogues’, including homocysteine and penicillamine, albeit with differing reaction rates relative to cysteine. In this case, a reported peptide inhibitor of the p53‐MDM2 and p53‐MDMX protein‐protein interaction was mutated to incorporate combinations of L‐Cys, D‐Cys, L‐Pen and hCys to subtly tune the binding affinity and target‐selectivity by altering the secondary structure.^[36]^ Perfluoroaromatics (no nitrogen atoms in the ring) tend to be cysteine‐selective in their reactivity, however, the crosslinking of lysine amines is made possible (Scheme 11) by increasing the electron‐deficiency through using activated perfluoroaryl reagents for example diperfluorophenyl sulfone (**48**).^[37]^

**Scheme 13:**
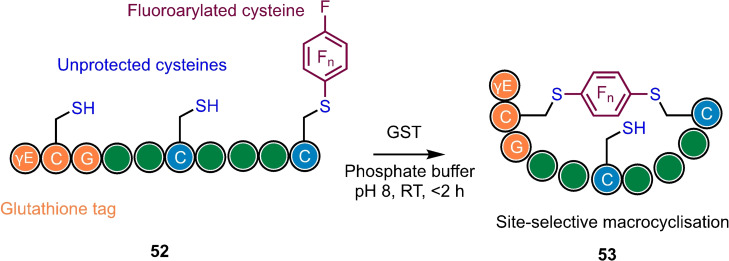
Site‐selective peptide macrocyclisation promoted by a glutathione tag in the presence of a glutathione *S*‐transferase. Note, peptide sequences shown are for illustrative purposes only. Based on Ref. [38].

Despite exhibiting excellent chemoselectivity, the site selectivity of fluorarylation is expected to be relatively non‐specific where the peptide system contains >2 cysteine residues lacking appropriate protecting groups or pre‐organisation. However, regiocontrol can be achieved by the incorporation of a ‘glutathione tag’ (γ‐Glu‐Cys‐Gly), that primes the peptide for regioselective fluoroaryl macrocyclisation mediated by a glutathione‐*S*‐transferase (GST) enzyme (Scheme 13).^[38]^

### 3.3 Reagents for site‐selective bioconjugation

Site‐selective protein modification allows biomolecules such as monoclonal antibodies with high‐target selectivity, to be conjugated with otherwise indiscriminately cytotoxic agents, to afford tissue‐targeted and selective agents for for example cancer treatment.^[39]^ Besides antibody‐drug conjugates, the ability to selectively attach bioactives to long‐lived plasma proteins or to attach fluorescent probes has transformed modern approaches to medicine.^[40]^

**Scheme 14:**
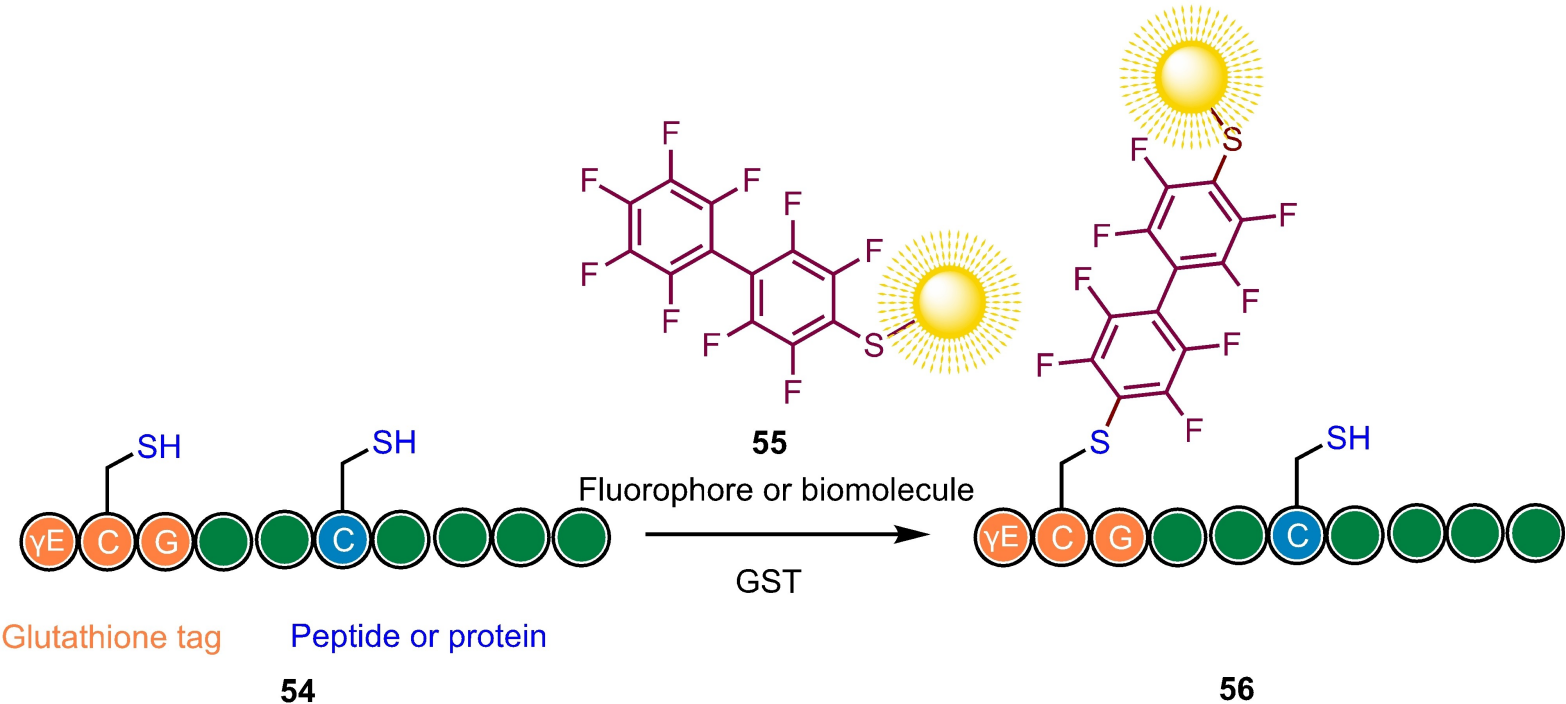
Site‐selective peptide or protein arylation as a means of bioconjugation with a prefunctionalised perfluoroaryl probe. Note, peptide sequences shown are for illustrative purposes only. Based on Ref. [42].

Protein labelling or bioconjugation is generally performed using cysteine‐selective reagents, such as maleimides.^[41]^ As discussed above, many perfluoroaromatics are highly susceptible to double substitution in a 1,4‐ or 4,4’‐ manner due to the increased stabilisation of the Meisenheimer‐Jackson intermediate by the sulfur atom from the first substitution. If only a single thiol nucleophile is available or a high excess of the perfluoroaryl reagent is used, a singly arylated product can be obtained (Scheme 14). This makes mono‐substituted perfluoroaromatics well suited as highly chemoselective bioconjugating reagents.

The main challenge associated with modification/conjugation of large polypeptides is reacting at the desired site when multiple chemically similar reactive handles are available. Another limitation of the methods described above is their requirement for an organic solvent, which is incompatible with biomolecules, whilst the reaction is also be hindered by the slow S_N_Ar reactivity of perfluoroaromatics in water. As we have seen earlier for perfluoroaryl macrocyclization, these challenges can be overcome by incorporating an N‐terminal glutathione (γ‐Glu‐Cys‐Gly) recognition‐sequence into peptides, whose monoarylation is specifically promoted at the glutathione cysteine in the presence of a glutathione‐S‐transferase enzyme.^[42]^

**Scheme 15:**
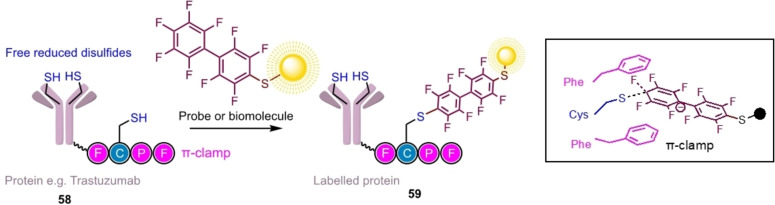
π‐clamp mediated site‐selective cysteine bioconjugation. The accelerated rate has been attributed to interactions between the Phe residues and the incoming perfluoroaromatic, as well as increasing Cys acidity. Based on Ref. [43].

Site‐/regioselectivity can also be obtained in an enzyme‐independent manner through the use of a ‘π‐clamp’ approach (Scheme 15). This incorporated a Phe‐Cys‐Pro‐Phe motif into a peptide, which promoted the selective reaction of the cysteine residue with perfluoroaromatic reagents (aqueous conditions, <1 h.^[43]^ Molecular dynamics (MD) modelling and density functional theory (DFT) proposed that the accelerated reaction rate at the FCPF motif was due to the π‐clamp providing a microenvironment that mimics an enzyme active‐site and tunes the reactivity of the cysteine by reducing the thiol pKa, recognising the perfluoroaromatic reaction partner and decreasing the enthalpy of activation for the S_*N*_Ar reaction.^[44]^

**Figure 3:**
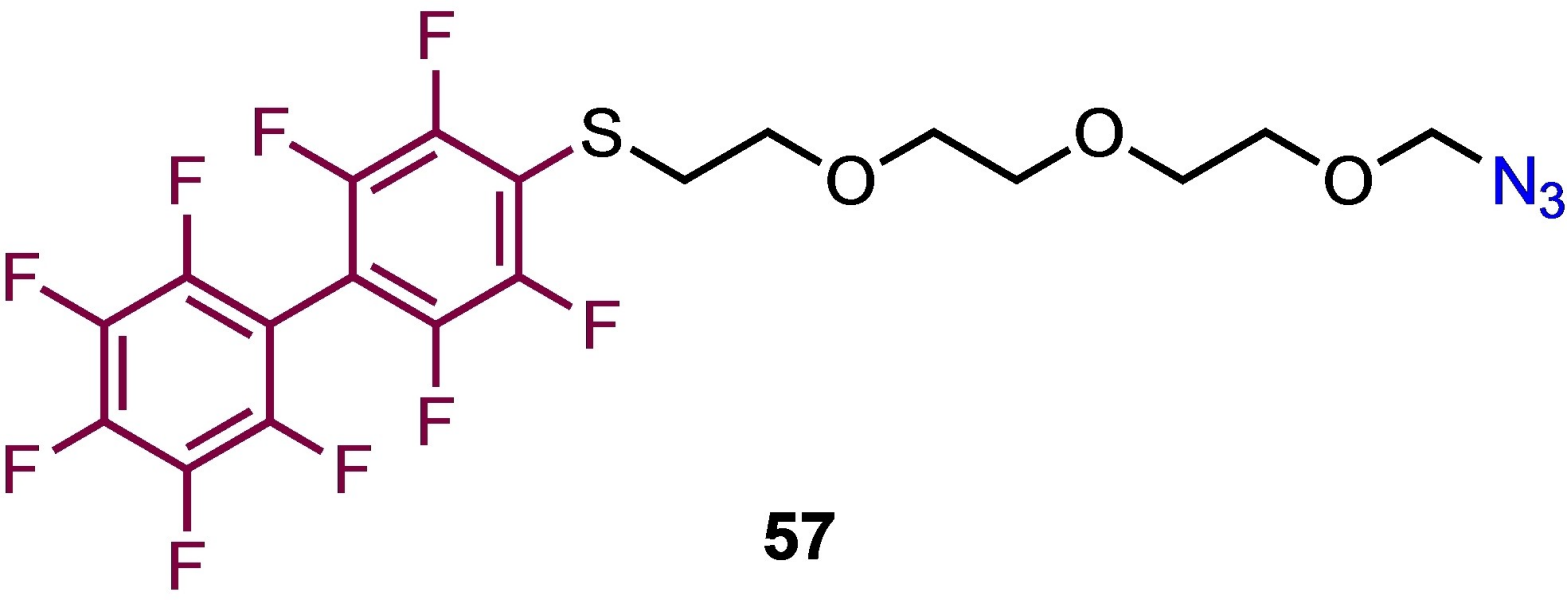
Molecular probe designed for efficient conjugation using the π‐clamp approach.

The π‐clamp approach was used to modify the C‐terminus of the heavy chains of trastuzumab (monoclonal antibody used in breast cancer treatment)^[45]^ enabling the synthesis of site‐specific antibody‐drug conjugates (Scheme 15) that retained binding affinity to their targets for selective killing of BT474 HER2‐positive breast cancer cells.^[43]^ The first non‐terminal location for the π‐clamp motif, was demonstrated in the investigation of the assembly of the major capsid protein of the JC polyomavirus, the causative agent of progressive multifocal leukoencephalopathy (PML).^[46]^ Recombinant truncated VP1 pentamers were prepared containing the π‐clamp motif (FCPF) in a non‐conserved and surface‐exposed mid‐sequence loop. This was found to be site‐selectively labelled using a modular probe **57** (Figure 3), in which nonafluorobiphenyl was linked by a triethyleneglycol spacer to an azide for CuAAC reaction with a variety of small molecule cargoes for example fluorophores.

The aqueous, biocompatible arylation reaction rate can also be controlled by adding salts, increasing rates by up to 4 orders of magnitude according to the Hoffmeister ions series, wherein for example ammonium sulfate is known to promote hydrophobic interactions and accelerates the bioconjugation of a model antibody in a concentration‐dependent manner.^[47]^

In a bid to understand the wider sequence effects on cysteine‐arylation and to discover cysteine‐containing polypeptide sequences with an intrinsic propensity to react with perfluoroaromatics, Evans et al. screened a library of random sequence ∼30‐mer peptides using mRNA display and high‐throughput sequencing.^[48]^ They identified a 29 amino acid peptide (MP01) (not containing the FCPF sequence) that had an intrinsically favourable rate of arylation (0.28 M^−1^ s^−1^ at pH 7.4 and room temperature), and therefore, underwent site‐selective cysteine arylation, with a water‐soluble perfluorodiphenyl sulfide‐based reagent, and afforded selective arylation in protein fusion constructs. Perfluoroaryl reagents were found to interact with MP01 (and related peptides) in an initial non‐covalent fashion (similarly to the π‐clamp), increasing the effective molarity and thus the reaction rate in analogy to enzymes.^[49]^ The arylation rate also appeared to be enhanced by the perfluoroaryl reagent‐inducing increased α‐helical character of the cysteine‐containing peptide indicating that the secondary structure of the nucleophilic species affects the reaction rate and site‐selectivity. Indeed, these observations appear to be general and applicable to a broad family of cysteine‐containing peptides, many of which adopt secondary structures (including β‐sheets) as part of the arylation process.^[50]^ As such, the authors proposed that the arylation reaction is dependent on the folded 3D structure of the peptide (and ablated by denaturation), in analogy to an enzyme active site.

### 3.4 Construction of multicyclic peptide systems

**Scheme 16:**
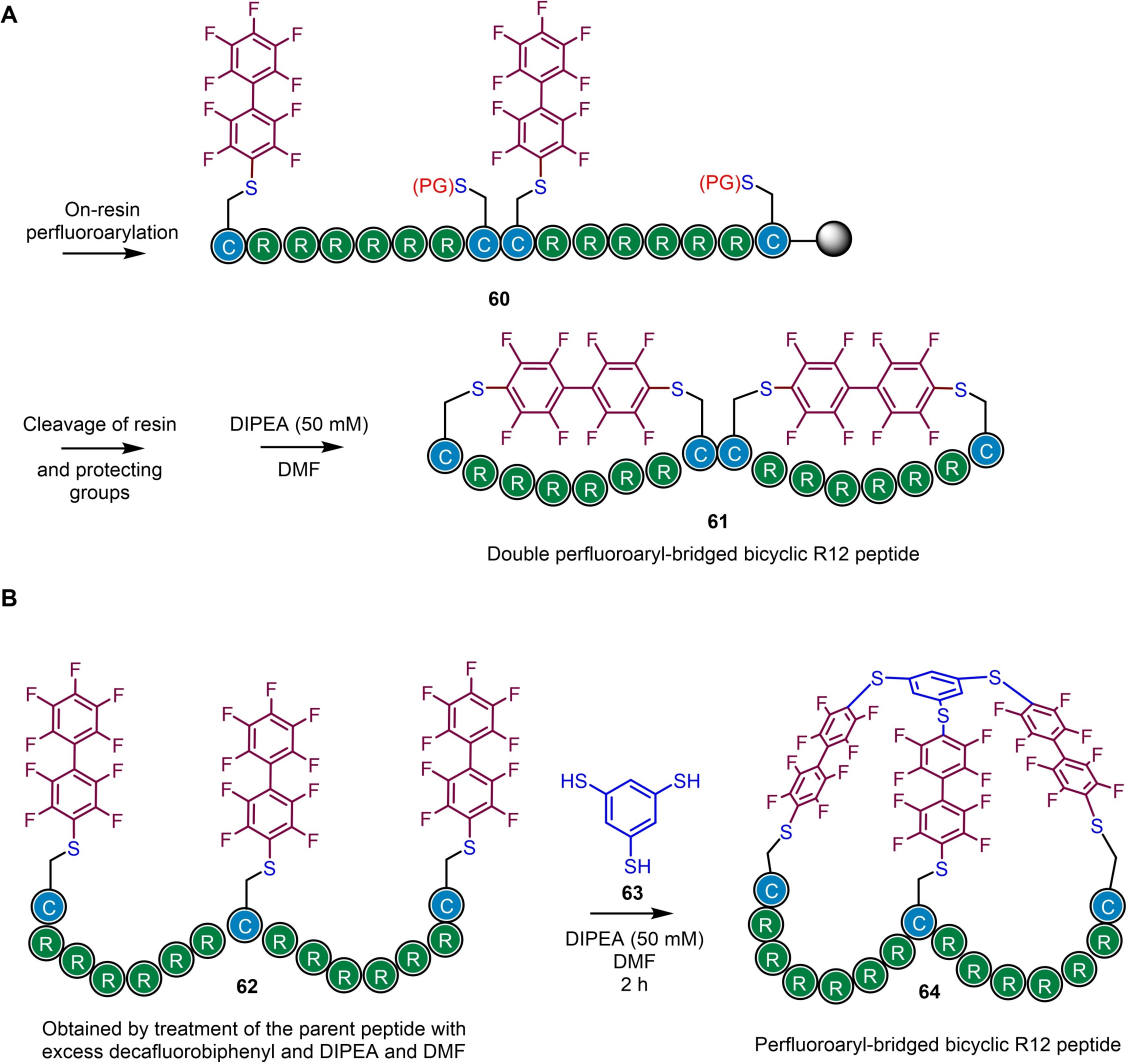
Approaches to bicyclic peptide systems *via* perfluoroylation and subsequent macrocyclization. Based on Ref. [52].

The construction of multicyclic peptide architectures through the reaction of tri‐/tetra‐cysteine containing peptides with tri‐/tetra‐bromomethyl benzene electrophiles and related reagents^[51]^ has revolutionised the screening of peptides displaying 3D protein epitope‐like features. Typically, these methods afford a mixture of regioisomers due to the identical reactivity of each electrophilic site. Bicyclic systems can conceptually be prepared by double perfluoroaryl‐macrocyclisation at two cysteine pairs (Scheme 16 (A)), however, this requires orthogonal protecting group shuffling to avoid multiple regioisomeric products. In an extension of earlier methodology, another way to obtain bicyclic peptides was achieved via perfluoroarylation of three separate cysteines with decafluorobiphenyl (mono‐substitution) to afford the tri‐arylated peptide, which is primed to undergo three secondary substitution reactions with 1,3,5‐benzenetrithiol (Scheme 16 (B)). In this study, bicyclic peptides exhibited higher proteolytic stability (trypsin) than monocyclic peptides and demonstrated promise as antisense oligonucleotide delivery agents.^[52]^

**Scheme 17:**
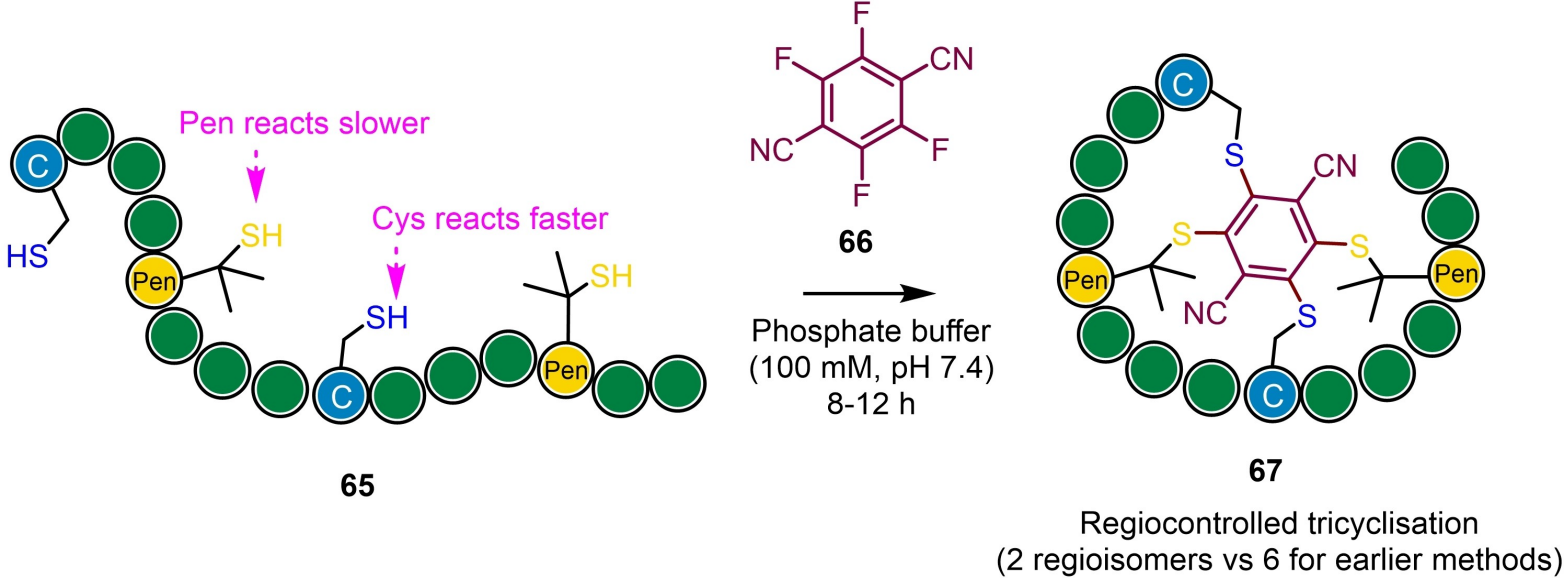
Precisely Regulated and Efficient (PROP) Locking of linear peptides containing differentially reactive nucleophiles (cysteine and penicillamine) into tricyclic architectures using 2,3,5,6‐tetrafluoroterephthalonitrile. Note, peptide sequences shown are for illustrative purposes only. Based on Ref. [53].

Polyfluoroaromatics can conceptually undergo multiple substitutions by peptide cysteines to afford for example tricyclic systems.^[40]^ However, due to the diminished reactivity of the scaffold reagent following the first two substitutions, subsequent substitutions either proceed very slowly or tend not to occur. Wu and co‐workers addressed this using 2,3,5,6‐tetrafluoroterephthalonitrile (**66**, Scheme 17).^[53]^ Under mild aqueous conditions, two *para*‐fluoride groups are substituted readily by thiols, followed by the slower substitution of the remaining two fluorides. The differential reactivity for the second pair of substitutions, as well as the lower nucleophilicity of penicillamine compared with cysteine (40‐fold difference due to steric hindrance by β‐dimethyl substituents), was exploited to afford sequential tetrasubstitutions, affording controlled access to redox‐stable tricyclic peptide systems containing two cysteine and two penicillamine residues with high regiocontrol (only 2 isomers possible).

## 4 Summary and Outlook

Here we have briefly detailed some of the many reasons that perfluoroaryl reagents occupy a prominent and growing space in peptide and protein chemistry. They have applications in peptide synthesis and bioconjugation, as well as potential applications in tuning the properties of bioactive synthetic peptides. The use of pentafluorophenyl esters of amino acids for peptide synthesis is uncommon, however, the use of the preformed activated esters provides the advantages of rapid coupling reactions and the avoidance of bringing the growing peptide chain into contact with the activating reagent, to reduce side reactions.^[19]^ This arguably provides better atom economy as compared with excessive equivalents of activator and additives. The clear disadvantage is the need to prepare the pentafluorophenyl ester in advance, particularly for any non‐standard or synthesised amino acids.

In many ways perfluoroarylation bears the hallmark of a ‘click’ reaction, being rapid, quantitative and having a wide functional group tolerance. Crosslinking of disulfides with perfluoroaromatic reagents for example, hexafluorobenzene gives predictable, highly regioselective substitution products due to the directing effect of the α‐carbanion‐stabilising sulfur atom from the initial substitution in the Meisenheimer intermediate. They can be tuned to react with nitrogen and oxygen‐based nucleophiles to afford broader applications compared with standard reagents that tend to be highly specific to a particular nucleophile for example, maleimides, sulfonyl fluorides or N‐hydroxysuccinamide esters. In comparison with other bioconjugation and crosslinking reagents, in general perfluoroaromatics do not require the use of unnatural amino acids containing for example, azides, alkynes or alkenes, which can be expensive to purchase or tedious to synthesise. Perfluoroaromatics react with natural amino acids to form stable and non‐reversible conjugates under physiological conditions, unlike other reagents, such as maleimides.^[54]^ Advantageously, perfluoroaromatics afford the distinct ability to monitor reactions and to identify substitution patterns around the aromatic ring using ^19^F NMR.^[35]^

In general, perfluoroaromatic bioconjugation can enhance the pharmacokinetic properties for example, serum stability and conformational stability through stapling of bioactive synthetic peptides (many examples above). However, perfluoroarylation can add significant lipophilicity to a biomolecule and may affect its solubility or cellular penetration in the case of for example, stapled or macrocyclic peptides, albeit, this probably is more dependent on the physicochemical properties of the starting peptide. Crosslinking of native disulfides can also introduce perturbation of native interactions and loss of pharmacological activity,^[55]^ which may be attributed to steric or conformational deformation of the peptide, albeit this can be also true of competitor approaches. Many simple perfluoroaromatics for example, hexafluorobenzene, decafluorobiphenyl can have poor solubility in water, requiring the use of organic co‐solvents, which may be incompatible with biomolecules, or the augmentation of the reagent to include water‐solubilising motifs (e. g., sulfones),^[56]^ which can also enhance the reaction rates in water. Indeed, there are now many perfluoroaromatic derivatives reported in the literature that have improved reactivity profiles, broader nucleophile reactivity and compatibility with aqueous media.

From a reactivity point of view, mono‐perfluoroarylation can be difficult to achieve when there are multiple reactive nucleophiles. This can be mitigated by using a large excess of perfluoroaromatic reagent or the inclusion of a heteroatom as in pentafluoropyridine. This increases the rate of mono‐substitution but the subsequent ‘*para’*‐substitution cannot occur. In practice, disubstituted perfluoroaromatics do not tend to undergo further substitution by thiols under mild/physiological conditions. However, as discussed above, this can potentially happen with more highly activated systems, increasing their metabolic liability, meaning that they may be subject to further conjugation with for example glutathione in vivo and may accelerate their degradation/excretion from the body.

Given that peptides can be readily and site‐selectively modified at a late stage by perfluoro(hetero)aryl reagents, this will provide new opportunities for medicine and healthcare in academia and pharma. The wide scope of nucleophiles that can be used in combination with perfluoroaryls also lends them to high‐throughput screening applications. For example, using complex mixtures of nucleophiles to rapidly generate compound libraries at the early drug discovery stage; or different size macrocycle screening are immediate opportunities. Given the reversibility of tyrosine perfluoroarylation, this can be optimised and exploited as a prodrug strategy to increase for example cell membrane permeability or to allow access to the BBB. Glimpses of the possible ability of fluoroarylation, either directly, or as an intermediary, to modulate ‘drug‐like’ properties, such as blood serum stability and blood‐brain barrier penetration is likely to promote a significant new tool for lead‐optimisation. Reactivity‐tuned fluoroarylated peptides may afford new warheads for highly targeted covalently reactive enzyme inhibitors, which have risen to prominence, specifically in the protein kinases field and may be employed in the emerging covalent PROTACs area. Given their biocompatibility, fluoroaromatic probes may also find applications as diagnostic agents for *ex vivo* ^19^F NMR analysis of primary patient samples to monitor biomarkers of disease. Moreover, [^18^F]‐fluoroaromatics may provide a new strategy for radiolabelling of peptides and proteins for personalised PET tracers, given their rapid and clean reactivity, which is well suited to the short radiochemical half‐life of ^18^F.^[57]^

From a purely chemical synthesis perspective the use of perfluoroaryl reagents offers an exciting avenue to deliver new molecular functionality. The ease and simplicity with which perfluoroaryl reagents can link together disparate molecular units gives these reagents a distinct advantage over more complex methodologies such as cross coupling. The use of these reagents for molecular linkage bares similarity to the use of copper‐catalysed azide‐alkyne cycloadditions (CuAAC) to access 1,2,3‐triazoles in “click chemistry” as both are quick and efficient linkage manifolds. Being able to exploit this reactivity in combination with the ability for these reactions to be reversed (not possible with CuAAC) could lead perfluoroaryl reagents to become a valuable tool in dynamic molecular linkage.

In conclusion, perfluoroaromatics and perfluoroheteroaromatic regents have been demonstrated to allow access to modified peptides and as highly efficient bioconjugation reagents. This area is an exciting and rapidly growing one with many future avenues for further exploitation. These reagents offer a new and easy to use toolbox to both peptide and synthetic chemists for molecular diversification and modification.

5

## Biographical Information

*Will Brittain is a Leverhulme Trust Early Career Fellow and Assistant Professor of Research at Durham University. He completed his PhD (awarded 2018) at the University of Birmingham with Professor John Fossey with an international placement with Professor Eric Anslyn at the University of Texas at Austin (2015) before moving to Durham to take up a PDRA position with Professor Steven Cobb from 2017–2020. He was awarded a Leverhulme Early Career Fellowship in 2020 to begin his independent career. His research interests include organofluorine chemistry, triazole synthesis and the application of peptides and peptidomimetics to catalysis. He was recently elected to the RSC Fluorine Interest Group Committee*.

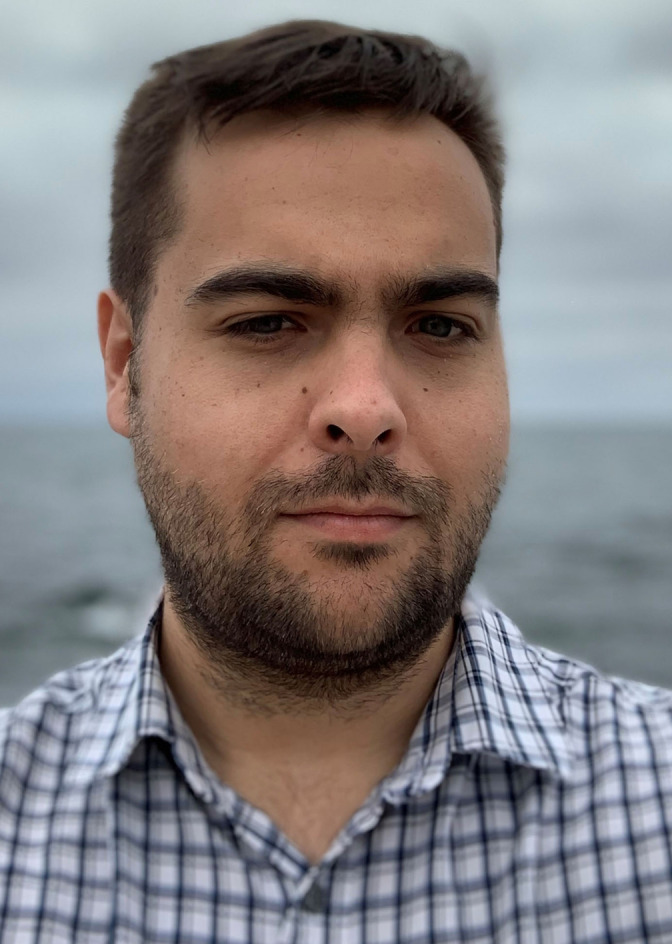

## Biographical Information

*Chris Coxon is Senior Lecturer in Medicinal Chemistry in the EaStChem School of Chemistry at University of Edinburgh, UK. His research focusses on peptide drug discovery and using organofluorine for peptide augmentation and tuning of physicochemical and pharmacokinetic properties. In 2010 he completed his PhD at The Northern Institute for Cancer Research, Newcastle University, before postdoctoral appointments at Durham University from 2010–2013. He previously held academic positions at Liverpool John Moores University and Heriot‐Watt University (2013‐2021). He is a panel member of the RSC Protein & Peptide Science Group (PPSG) and Director and co‐founder of Pepmotec Ltd*.

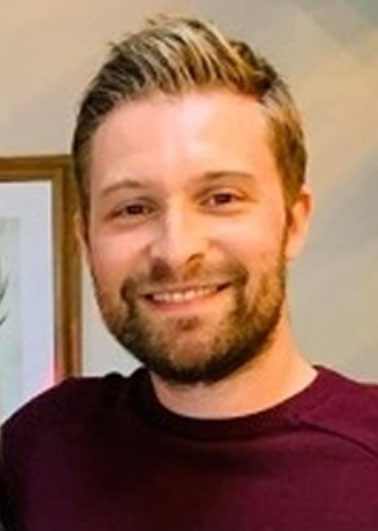

## Data Availability

Data sharing is not applicable to this article as no new data were created or analyzed in this study.
